# High heterotrophic counts in potable water and antimicrobial resistance among indicator organisms in two peri-urban communities of Karachi, Pakistan

**DOI:** 10.1186/s13104-018-3461-z

**Published:** 2018-06-04

**Authors:** Sadia Shakoor, Imran Ahmed, Saima Mukhtiar, Israr Ahmed, Farzeen Hirani, Shazia Sultana, Rumina Hasan

**Affiliations:** 1 Pathology & Laboratory Medicine, Aga Khan University, Karachi, Pakistan; 2 Pediatrics & Child Health, Aga Khan University, Karachi, Pakistan

**Keywords:** Potable water, Household, Heterotrophic plate count, Peri-urban, Antimicrobial resistance

## Abstract

**Objective:**

Fecal contamination of potable water leads to unsafe water supply. Although many urban areas of large metropolitan cities receive safe water, peri-urban areas are often not monitored by public health authorities and water supply and quality remain unknown. We assessed microbiological quality and rates of antimicrobial resistance in viable indicator bacteria in two peri-urban communities of Karachi, Pakistan. Water samples were collected over 5 months (October 2015 to February 2016) from these peri-urban communities and samples were processed for microbiological quality as per Standing Committee of Analysts, United Kingdom and World Health Organization guidelines and criteria for drinking water.

**Results:**

Both communities received unimproved water. Potable water samples collected from 100 households showed that 96% of samples were unsafe for consumption. Extended spectrum beta lactamases production was found in 29.2% of fecal indicator organisms (coliforms). Use of unimproved water sources and unsafe potable water quality in peri-urban Karachi deserve immediate attention and upgrade. The study is instrumental in attracting the attention of authorities to the state of water resources in peri-urban communities in Karachi with a view to influence improvement of services and effects on human health.

## Introduction

Safe drinking water is a basic human right [1] and sustainable development goal (SDG) 6 endorses efforts toward provision of safe and affordable drinking water for all by 2030 [2]. Lack of access to safe drinking water negatively impacts human health and results in childhood illness, infectious outbreaks, food security, and livelihood [3]. Deleterious health effects of water shortage are observed in underserved communities, many of which are located around populated megacities. Karachi is a coastal megacity in southern Pakistan with a population of 16 million, and an annual growth rate of 6% [4]. Karachi’s urban geography is surrounded by a large peri-urban agglomerate. A recent review identified Karachi as one of four highly vulnerable megacities with regard to water supply and urban water security [5].

Peri-urban communities are in transition between urban and rural settings. Supply and quality of natural resources such as water are often inadequate in peri-urban communities [6] around expanding megacities such as Karachi. Piped water may not always be available and consumers often rely on groundwater sources, draw water from distribution lines through suction pumps, or purchase water from vendors [7]. Since only municipal piped water may be chlorinated, the quality of water reaching peri-urban areas suffers from high levels of contamination. For similar reasons, measures to monitor and maintain microbiological standards of water overlook peri-urban areas. Therefore, little is known about water sources, quantity, and quality of potable water in these areas of Karachi.

Data from the Global Enteric Multicenter Study (GEMS) showed a high burden of childhood diarrhea in peri-urban Bin Qasim Town of Karachi, and extremely limited access to improved water sources [8]. Many pathogens in the study with high attributable fractions for childhood diarrhea are water-borne, suggesting that microbiological quality of potable water must be examined. Monitoring of microbiological water quality is a prerequisite for risk benefit analysis to define practical safety limits for drinking water and human health impact. In this non-hypothesis, descriptive study, we have assessed potable water sources and microbiological quality in the peri-urban communities of Bin Qasim town of Karachi. The data provides insight into existing water sources and contamination levels with the ultimate purpose of identifying health risks and highlighting opportunities for improvement of health outcomes of peri-urban communities in Karachi.

## Main text

### Methods

#### Study sites

**Fig. 1:**
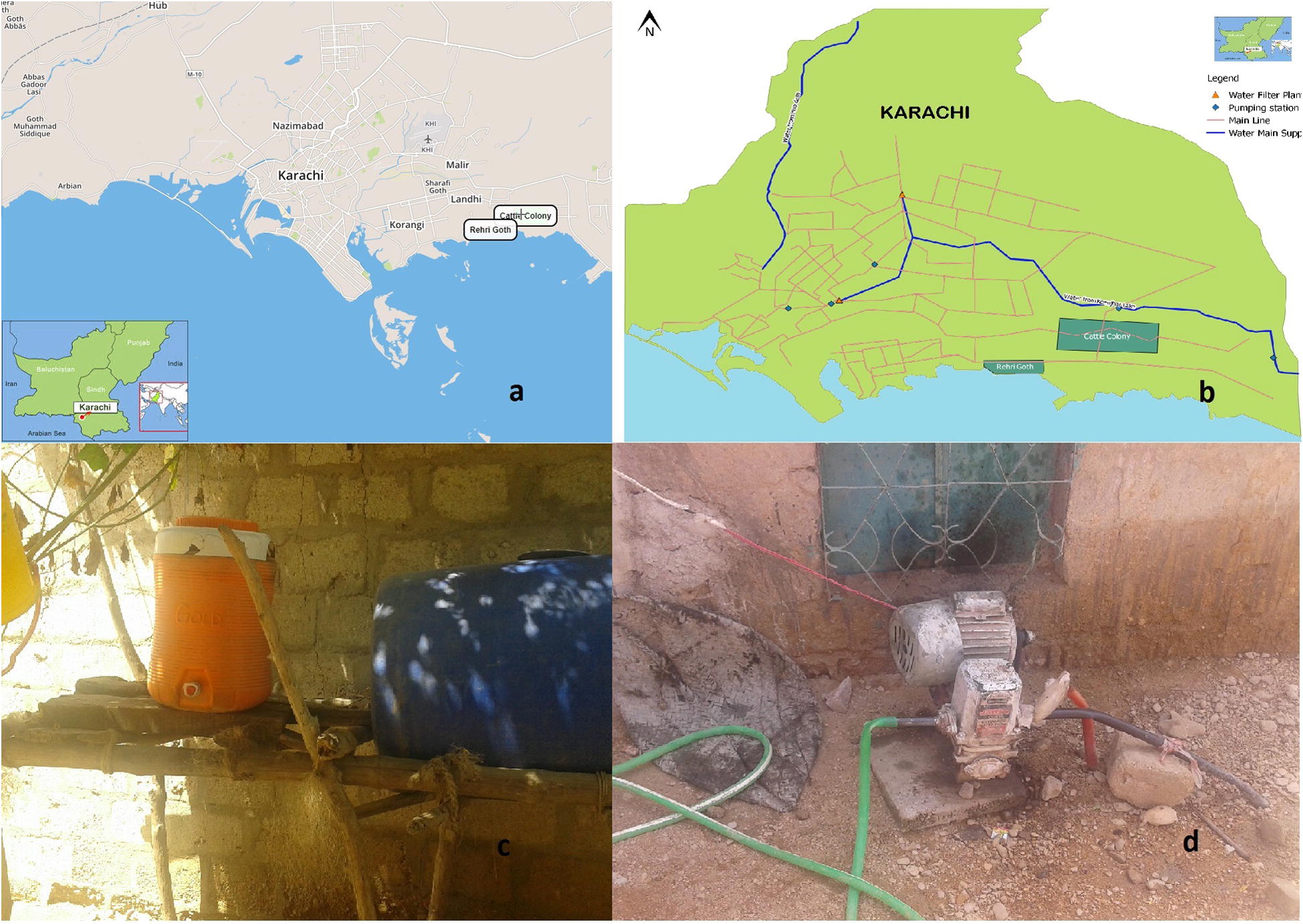
Study areas in Karachi and water supply and consumption; **a** Map of Cattle Colony and Rehri Goth in the Bin Qasim Town of Karachi (Insets show location of Karachi within Pakistan and Pakistan in South Asia); **b** water distribution system in Karachi showing piped water supply to study areas from a natural lake east of Karachi; **c** water storage tanks used in some households; and **d** pressure booster pumps used by consumers to overcome low pressure and intermittent supply of piped water

Cattle Colony (CC) and Rehri Goth (RG) are peri-urban localities around cattle-farming and fishing communities, respectively, in the Bin Qasim town of Karachi (Fig. 1a). The sites were included in the Global Enteric Multicenter Study as surveillance sites for diarrhea in children and have an established Health and Demographic Surveillance System. The sites receive piped water supply from the Kinjhar lake system (Fig. 1b).

#### Sampling methods

Household water (500 mL) was collected in sterile plastic containers with sodium thiosulfate from 50 households each in CC and RG, from October 2015 to February 2016. Water was collected directly (without prior run-off) from sources of consumption in households (Fig. 1c). Samples were transported at room temperature within 4 h of collection to the Aga Khan University clinical microbiology laboratory for processing.

#### Microbiological methods and susceptibility testing

Water samples were processed within 24 h of receipt at the laboratory. Turbidity was measured by the turbidity tube method [9] and results were recorded in turbidity units (TUs). For heterotrophic plate counts (HPCs), 1 mL of water was added to 19 mL of yeast extract agar (Oxoid) and placed at 37 °C and results were read at 44 ± 4 h as recommended by the Standing Committee of Analysts, United Kingdom (SCA, UK) guidelines [10–12]. HPC counts were read with a colony counter on the 100 mm petri plate, the count limit for which was 5700 colony forming units (CFU)/mL. For coliforms and thermotolerant *E. coli*, 100 mL of water was filtered through a 47 mm 0.45 Millipore filter (Merck) and the membrane placed onto Membrane lauryl sulfate agar (Conda Laboratories). Two paired volumes were inoculated onto MLSA and placed at 30 °C for 4 h followed by incubation for 14 h at 37 °C (for presumptive coliform growth) and 44 °C (for presumptive *E. coli* growth). Presence of coliforms and *E. coli* was recorded and counts were not performed. For confirmation of coliforms and *E. coli*, oxidase test and a combination of sulfide, and indole production were used, respectively. For recovery of *Salmonella* and *Shigella*, buffered peptone water was set up at 37 °C for 24 h, followed by Rappaport Vassiliadis broth (Oxoid) enrichment and culture on xylose lysine deoxycholate agar (Oxoid) to recover colored colonies (pink-red-yellow with or without black centers).

Overall microbiological quality was considered acceptable if no coliforms and/or *E. coli* were recovered from 100 mL of sample [13]. Since > 500 CFU/mL of HPC inhibit recovery of coliforms and *E. coli* on lactose containing media, HPC counts exceeding 500 CFU/mL were also considered unacceptable [14], irrespective of the recovery of coliforms and thermotolerant *E. coli*.

A subset of randomly selected coliforms and *E. coli* were subjected to antimicrobial susceptibility testing. Briefly, identification was confirmed with Activity profile Index (API) 20 E (bioMereiux), and colonies of selected isolates were suspended in sterile normal saline to achieve a turbidity of 0.5 McFarland, and tested as per manufacturer’s recommendations on the Gram negative Vitek (bioMereiux) card. Antibiotics tested included piperacillin, ceftazidime, ciprofloxacin, meropenem, and trimethoprim-sulfamethoxazole. Extended Spectrum Beta Lactamase (ESBL) production was determined by the Vitek Advanced Expert System (AES) through determination of susceptibility against third generation cephalosporins and ceftazidime.

#### Data entry and analysis

All data was entered and frequencies and medians were calculated using SPSS v19.0 (IBM). Median HPCs were compared using Mann–Whitney U test.

### Results

#### Household characteristics

Of 100 households included in the study, piped water supply was available in 55% (n = 55; 38 in CC and 17 in RG), water was purchased from contractual tanker hydrant suppliers in 36% (n = 36; 8 in CC and 28 in RG), and groundwater was used in 9% (n = 9; 4 in CC and 5 in RG). Households with piped water supply used pressure boosting pumps to ensure continuous supply of water (Fig. 1d). Neither boiling nor chlorination were used in any of the households; 2 households in CC filtered piped water. More than half (55%; n = 55, 40 in CC and 15 in RG) used vessels for storage of drinking water; others used these directly from the source.

#### Turbidity and microbiological acceptability

Turbidity was < 5 TUs in 67% of samples, 10 TU in 7% of samples, and > 10 but < 20 TU in 26 samples, with visible particulate matter.

Microbiological quality was unacceptable in 96% of samples. Heterotrophic Plate Counts (HPCs) and recovery of coliforms and thermotolerant *E. coli* from samples in CC and RG are shown in Table 1. Very high HPCs inhibited growth of coliforms and *E. coli* in 4% of samples, but these were considered unacceptable based on their high HPCs. *Salmonella* spp. and *Shigella* spp. were not recovered from any samples. Median HPCs did not significantly differ between piped and purchased water sources (p = 0.1).

**Table 1:** Proportion of piped water supply source and microbiological acceptability of potable water samples from households in Cattle Colony (CC) and Rehri Goth (RG) peri-urban communities of Karachi, Pakistan

	CC	RG	Total
% Piped source	76 (n = 38)	34 (n = 17)	55 (n = 55)
Median HPC (IQR)	4004 CFU/mL (1647–5700)	5700 CFU/mL (2779–5700)	5700 (1781.5–5700)
% Microbiologically unacceptable	92 (n = 46)	100 (n = 50)	96 (n = 96)
% Samples with coliforms isolated	90 (n = 45)	94 (n = 47)	92 (n = 92)
% Samples with thermotolerant *E. coli* isolated	26 (n = 13)	44 (n = 22)	35 (n = 35)
	50	50	100

#### Antimicrobial resistance

Antimicrobial resistance was further evaluated in randomly selected 113 indicator organisms recovered from 100 samples. Resistance rates against common antibiotics are shown in Table 2. Data presented show a higher resistance against cephalosporins, fluoroquinolones, and cotrimoxazole in *E. coli* than in other coliforms. Carbapenem resistance was not detected.

**Table 2:** Resistance to third generation cephalosporins, ciprofloxacin, and trimethoprim sulfamethoxazole among randomly selected organisms cultured from potable water in peri-urban households

Organism	Number	% Resistant (n)			Total
		Ceftazidime	Ciprofloxacin	Trimethoprim/sulfamethoxazole	
*E. coli*	37	43.2 (16)	10.8 (4)	29.7 (11)	37
*Enterobacter* spp.	17	5.9 (1)	0	29.4 (5)	17
*Klebsiella pneumoniae*	56	26.8 (15)	0	8.9 (5)	56
*Citrobacter* spp.	3	33.3 (1)	0	0	3
Total	113	29.2 (33)	3.5 (4)	18.6 (21)	113

### Discussion

Results from our study show a very high recovery rate of heterotrophic organisms from household potable water in peri-urban Karachi, with a high fecal contamination rate. Governance and infrastructural challenges in peri-urban areas hinder sustainable water supply and management [6], which in turn lead to unsafe microbiological quality of potable water. Our results reflect these underlying challenges in peri-urban community dwellings of Karachi. Routine monitoring of peri-urban water supply systems is also not carried out, and resulting high levels of microbial counts may lead to adverse human health outcomes. These results therefore indicate that peri-urban areas are in urgent need of attention with respect to water supply, safety and sustainability.

Our results also show the multiplicity of methods employed to obtain water. Piped water was not uniformly accessible, and where available, had insufficient hydraulic pressure and was interrupted, prompting residents to employ pressure booster pumps (Fig. 1d). Negative pressures in piped systems draw water from contaminated groundwater and even leaky sewage lines [15]. Furthermore, use of water lifting and booster pumps further increase contamination through ingress of air and surge effects.

Previous assessments of water quality in urban Karachi have established high levels of microbial contamination. The 2014 Multiple Indicator Cluster Survey (MICS) [16] demonstrated that 74.9% of households were using contaminated water with coliform counts of > 1 CFU/mL. In our study, rates are likely higher due to peri-urban location. Moreover, our interpretation of microbiological quality also included high HPCs as an indicator of inhibited coliform growth [17]. Since the MICS survey and the Pakistan National Quality Standards (NQS) for Drinking Water [18] did not consider HPCs when evaluating water quality, the proportion of unsafe water quality may be underestimated. In our study, we identified 4 samples with high HPCs and no coliform yield, suggesting that such instances are likely to be encountered with a finite frequency. We therefore recommend that NQS should include HPCs and consider high HPC counts (> 500 CFU/mL) to indicate water is unsafe for consumption.

High levels of HPCs have also been correlated with seasonal changes, increasing in summer months and during the wet season [19]. We collected water samples in winter months with little or no rain in Karachi, therefore our results are not likely to be high owing to seasonal changes.

While point-of-use disinfection remains a viable option safe potable use, technologies used may not be accessible, affordable, reliable, or even safe [20]. Techniques such as boiling are inherently unsafe due to the risk of burns [21], especially in crowded households with large volume needs. Chlorination of water by end users remains a useful strategy, is affordable, but consistent use requires a change in behavior and practices which need long-term investments in community education [22].

Antimicrobial resistance in the environment is an emerging concern. Higher rates of resistance among thermotolerant *E. coli* than in other pathogens, which may have multiple other sources than human and animal guts (such as industrial wastewater, vegetable dead matter, and soil) [23], reflect antibiotic resistance among the human and animal populations as being the main drivers for the observed resistance in water. This suggestion requires further confirmation through culture and molecular studies. Our results also show low prevalence of extended spectrum beta lactamase mechanisms (as indicated by third generation cephalosporin resistance) among indicator coliforms. Since many bacteria may not be recovered on culture, detection of antibiotic resistance genes (ARGs) may be a better indicator of prevalent transferrable resistant genes in water [24]. Although overall trends in quantity of antibiotic resistant bacteria may correlate with ARGs [25], the potential role of drinking water in transmission and acquisition of antibiotic resistance in humans remains unknown.

### Conclusions

Lack of adequate water supply and unsafe water has important implications on citizenship and social engagement [26] of communities. We have highlighted the problem of microbiologically unsafe potable water among peri-urban households in Karachi, which can have harmful physiological and psychological impact on the health of peri-urban dwellers. Urgent operational preventive measures to allow adequate resource allocation, community engagement and educational measures at the household level are necessary to avoid these health risks.

## Limitations

Our study did not focus on variations in rainfall and seasonal effects, dissolved organic matter, and chemical composition of water, all of which impact water quality and may also impact risk to human health. However, our findings remain an important indicator of the need for infrastructural as well as educational reforms to improve water quality to prevent enteric infections.

Both poor water quality and presence of indicators of human fecal contamination strongly suggest presence of diarrheal pathogens which could not be recovered due to use of only conventional methods. In addition, overwhelming numbers of bacteria as indicated by very high HPCs also interfere with pathogen detection and coliform isolation [17].

## Abbreviations

API: activity profile index
ARG: antibiotic resistance gene
C: centigrade
CC: Cattle Colony (Karachi peri-urban study area)
CFU: colony forming units
GEMS: Global Enteric Multicenter Study
*E.coli*: *Escherichia coli*
ESBL: extended spectrum beta lactamase
HPC: heterotrophic plate count
IQR: interquartile range
MICS: Multiple Indicator Cluster Survey
mL: milliliter
NQS: National Quality Standards
RG: Rehri Goth (Karachi peri-urban study area)
SCA: Standing Committee of Analysts
SDG: sustainable development goals
spp: species
TU: turbidity unit
UK: United Kingdom
WHO: World Health Organization
